# Retraction Note: Therapeutic effect of arthrocnemum machrostachyum methanolic extract on Ehrlich solid tumor in mice

**DOI:** 10.1186/s12906-025-05172-7

**Published:** 2025-11-05

**Authors:** Zeina W. Sharawi

**Affiliations:** Biological Sciences Department, Faculty of Sciences, King Abdulaziz University, P.O Box 80203, Jeddah, 21589 Saudi Arabia

**Retraction Note: BMC Complementary Medicine and Therapies 20, 153 (2020)**

**https://doi.org/10.1186/s12906-020-02947-y**

The Editor has retracted this article. Following publication, concerns were raised about the images presented in Fig. 3, specifically:

1. Figure 3C and D appear to share some repetitive elements;
2. Figure 3D in this article and Fig. 3d from a retracted article [1] appear to overlap.

The author was contacted to clarify the issue but has not provided satisfactory explanations and raw data to address the concerns raised. The Editor therefore no longer has confidence in the presented data.

Zeina W. Sharawi disagrees with the retraction.
